# Engineering an AlkS‐PalkB Transcription Factor‐Based Biosensor With Improved Sensitivity to Isobutanol and Other Short‐Chain Alcohols

**DOI:** 10.1111/1751-7915.70288

**Published:** 2025-12-25

**Authors:** Jiaming Chen, Xizi Wang, Can Zhu, Stefanie Frank, Jian Hao, Frank Baganz

**Affiliations:** ^1^ Department of Biochemical Engineering University College London London UK; ^2^ Lab of Biorefinery Shanghai Advanced Research Institute, Chinese Academy of Sciences Shanghai People's Republic of China

**Keywords:** biosensor, high‐throughput screening, isobutanol, synthetic biology, transcription factor‐based system

## Abstract

Isobutanol, a promising biofuel with higher energy content than ethanol, presents a sustainable alternative through biosynthesis. However, enhancing yield remains challenging due to the inefficiencies in microbial synthesis. This study introduces a transcription factor‐based biosensor using the AlkS‐PalkB system in *Escherichia coli* , which correlates green fluorescence with isobutanol concentration. Employing directed evolution, we modified AlkS to detect isobutanol, significantly improving biosensor specificity. Initial modifications increased the dynamic response from non‐detectable to a 2.60‐fold change. Subsequent optimisations through site‐directed mutagenesis and promoter engineering further enhanced this response to a 5.56‐fold change, equivalent to a 114% increase. Although engineered for isobutanol detection with high sensitivity, the engineered biosensor retains responsiveness to several short‐chain alcohols. This biosensor provides a foundation for high‐throughput screening of isobutanol and other short‐chain alcohol‐producing strains, though additional improvements in selectivity and operating range may be required for efficient implementation.

## Graphical abstract

Employing directed evolution, we modified AlkS to detect isobutanol, significantly improving biosensor specificity. Subsequent optimisations through site‐directed mutagenesis and promoter engineering further enhanced the dynamic range to 5.56‐fold change, equivalent to a 114% increase. This biosensor provides a foundation for high‐throughput screening of isobutanol and other short‐chain alcohol‐producing strains.

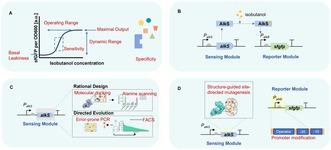

**Abbreviations**
A/Ala: alanine
cryo‐EM: cryo‐electron microscopy
D/Asp: aspartic acid
DBTL: design‐build‐test‐learn
DMSO: dimethyl sulfoxide (DMSO)
dsDNA: double‐stranded DNA

*E. coli*

:
*Escherichia coli*

E/Glu: glutamic acid
epPCR: error‐prone PCR
F/Phe: phenylalanine
FACS: fluorescence‐activated cell sorting
FSC‐H: forward scatter height
HTH: helix‐turn‐helix
HTS: high‐throughput screening
I/Ile: isoleucine
L/Leu: leucine
M/Met: methionine
N/Asn: asparagine

*P. putida*

:
*Pseudomonas putida*

PBS: phosphate‐buffered saline
Q/Gln: glutamine
R/Arg: arginine
S/Ser: serine
SSC‐H: side scatter height
T/Thr: threonine
TEV: tobacco etch virus protease
TF(s): transcription factor(s)
V/Val: valine
WCB: whole‐cell‐based biosensor
Y/Tyr: tyrosine

## 1 Introduction

Isobutanol, a short‐chain aliphatic alcohol, presents a superior alternative to ethanol as a sustainable biofuel due to its higher energy content and compatibility with existing fuel infrastructure. Unlike ethanol, isobutanol can be blended at higher concentrations without requiring modifications to existing engines or distribution networks, offering a seamless transition from fossil fuels. Moreover, its low vapour pressure reduces volatility, diminishing environmental and health risks associated with fuel evaporation (Liu et al. 2016; Nawab et al. 2024; Ni et al. 2023). These attributes not only promote isobutanol as a biofuel but also enhance its utility in the production of pharmaceuticals and fine chemicals, demonstrating its broad applicability across various industries (Chen and Patel 2012; Erickson et al. 2012; Liao et al. 2019; Lisovskii et al. 2002; Maketov et al. 1993).

Despite its potential, the production of bio‐based isobutanol faces significant challenges, primarily due to the limited yields achievable through microbial synthesis. Established microbial pathways for isobutanol production exist; however, their scalability is constrained by inefficiencies in the currently available microbial strains (Atsumi et al. 2008). The core challenge is the need for rapid and effective screening methods to identify and optimise high‐yield isobutanol‐producing strains, a process that traditional microbiological methods cannot expedite due to their labour‐intensive nature.

Efforts to enhance the yield of bio‐based isobutanol have included enzyme engineering (Gu et al. 2017; Lee et al. 2012; Novak et al. 2020), metabolic pathway engineering (Atsumi et al. 2008; Ida et al. 2012; Li et al. 2012), and mutagenesis (Li et al. 2012). Given the extensive mutation libraries produced by mutagenesis and the relatively low probability of obtaining positive mutants, the implementation of a high‐throughput screening (HTS) strategy is crucial for enhancing the efficiency of detection. A well‐designed biosensor, comprising an analyte‐specific receptor, a biosensor interface, a transducer, and an output system, offers a powerful solution (Zeng et al. 2020). Biosensors can be categorised as enzyme‐based, affinity‐based, and whole‐cell‐based systems. The primary operational principle of a whole‐cell‐based biosensor (WCB), particularly within optical biosensors, involves utilising living microbial cells to detect specific substances in their environment (He et al. 2016). An in vivo transcription factor (TF)‐based biosensor employs a modified TF as its transducer. Typically used for gene activation or repression, a TF contains a binding site ready for interaction with its specific ligand. The binding between the agent and the target DNA induces a conformational change, thereby modulating the accessibility of RNA polymerase and influencing gene expression (He et al. 2016; Pham et al. 2022).

**FIGURE 1:**
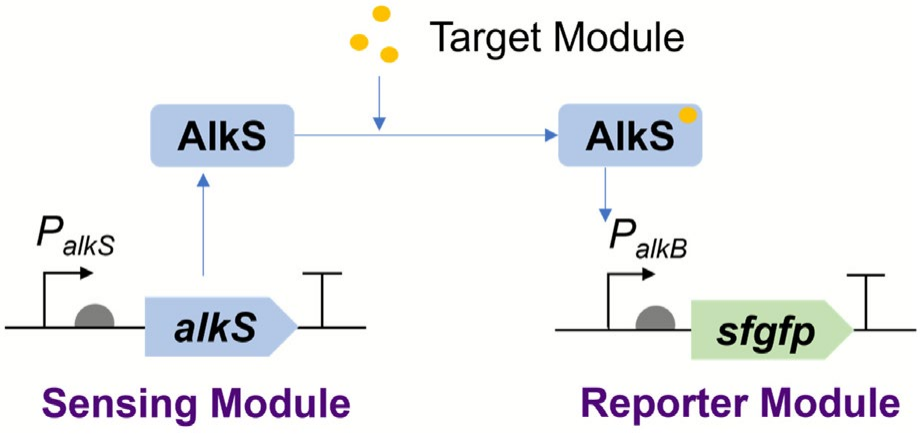
Schematic diagram of AlkS‐PalkB biosensor operation. The AlkS‐PalkB biosensor system utilises the transcription factor AlkS to detect the presence of specific alcohols. Upon binding of the target molecule to AlkS, the PalkB promoter is activated, triggering the transcription of a reporter gene (e.g., sfGFP). This results in a quantifiable fluorescence signal that correlates with analyte concentration, enabling rapid and sensitive detection.

Several TF‐based biosensors have been explored for isobutanol detection. For example, the BmoR‐Pbmo system has been reported to detect isobutanol over a wide concentration range (0–100 mM) (Yu, Chen, et al. 2019; Yu, Wang, et al. 2019). However, its specificity toward other alcohols has not been systematically tested, limiting conclusions about its suitability for high‐throughput screening. Additionally, the AlkS‐PalkB system has emerged as another promising candidate for detecting medium‐chain alkanes and alcohols. AlkS is a helix–turn–helix transcriptional activator originally characterised in *Pseudomonas putida* GPo1, where it regulates the alkane degradation pathway (Canosa et al. 2000; Fennewald et al. 1979; van Beilen et al. 2001). Upon binding of alkanes or short‐chain alcohols, AlkS undergoes conformational changes that improve its ability to recruit RNA polymerase and activate transcription from the PalkB promoter, thereby triggering reporter gene expression, such as sfGFP (Figure 1). The native expression of the pathway is regulated by the transcriptional activator AlkS. In the absence of inducers (short‐chain alcohol), only trace amounts of AlkS are produced from the *σ* ^S^‐dependent PalkS1 promoter, while the PalkS2 promoter remains inactive. Upon alkane entry into the cell, AlkS activates both the PalkB and PalkS2 promoters while continuing to repress PalkS. This feedback regulation ensures rapid AlkS accumulation in the presence of the inducer, while minimising unnecessary expression and cellular burden in its absence (Canosa et al. 2000). Previous studies have shown that replacing the PalkS promoter with constitutive promoters of varying strengths did not yield optimal performance. A weak constitutive promoter substantially reduced the maximum biosensor output, whereas a strong promoter produced similarly high fluorescence but resulted in undesirably high basal expression. The medium‐strength promoter generated a response profile comparable to that of the native PalkS circuit but exhibited higher basal activity and a lower maximum output. Therefore, the native PalkS promoter was selected for further research, as its intrinsic feedback regulation provides tight expression control, characterised by low basal activity and a high dynamic response, thereby ensuring optimal system performance (Bahls et al. 2022). Accordingly, we also employed the native PalkS promoter in our study. This mechanistic understanding guided the engineering strategy, targeting residues predicted to form the effector‐binding pocket and optimising the promoter architecture to improve transcriptional output in *E. coli* . Recent studies have demonstrated the feasibility of adapting this system for detecting branched chain alcohols, including isobutanol, although further engineering is required to enhance specificity and reduce cross‐reactivity. Although a previous study successfully constructed an isobutanol‐sensitive biosensor based on TF technology, it was noted that this biosensor exhibited leaky expression of the output signal at isobutanol concentrations below the detection threshold (Bahls et al. 2022).

Typically, naturally existing TF‐based biosensors do not meet the requirements for high‐throughput screening and thus require further optimisation. To be effective, biosensors should possess high specificity toward the target analyte to minimise false‐positive results. They should also detect specific analyte concentrations and exhibit a noticeable response range to differentiate effectively between varying analyte levels (Mahr and Frunzke 2016; Tellechea‐Luzardo et al. 2023). The performance of TF‐based biosensors can be optimised through strategies such as rational design and directed evolution. Their efficacy should be assessed based on key parameters like dynamic range, operating range, leakiness level, and sensitivity, which are often characterised using a Hill Curve (Dietrich et al. 2013; Flachbart et al. 2021; Hicks et al. 2020; Li et al. 2023; Sarnaik et al. 2020). For example, a recent study demonstrated that engineering ligand‐related exporters can shift the detection range of whole‐cell biosensors toward higher analyte concentrations, thus enabling more effective screening of high‐yield producer strains that accumulate large amounts of target molecules (Li et al. 2025). Moreover, advances in whole‐cell sensing platforms have expanded beyond conventional TF‐based sensors: for example, a recent study presented a broadly tuneable microbial biosensor platform in *Pseudomonas putida* that integrates fluorescent reporters with synthetic auxotrophies and growth‐coupled detection, enabling monitoring of structurally diverse chemicals in various screening formats (Hernández‐Sancho et al. 2024).

The aim of this research is to develop an AlkS‐PalkB transcription factor‐based biosensor embedded in *E. coli* , specifically tailored for HTS of isobutanol‐producing strains. The goal is to construct a leakage‐free biosensor capable of detecting isobutanol with high sensitivity and specificity, thereby significantly enhancing the efficiency of the screening process. To achieve this, AlkS was engineered through a combination of rational design and directed evolution to enhance its sensitivity and specificity toward isobutanol detection while minimising background leakage.

## 2 Materials and Methods

### 2.1 Bacterial Strains, Media, and Cultivation Conditions

All strains utilised in this study are summarised in Table S1.

For all plasmid construction, *E. coli* DH5α (New England Biolabs, Beverly, MA, USA) was used, and *E. coli* BW25113 was used for AlkS library screening and biosensor characterisation.

Glycerol stock cells were preserved at −80°C, containing 25% (v/v) glycerol. To isolate a single colony, a streak was made from the glycerol stock onto LB agar plates supplemented with appropriate antibiotics. This was followed by inoculation into 10 mL of Luria‐Bertani (LB) broth (Sigma‐Aldrich, St. Louis, MO, USA) containing the corresponding antibiotics in a 50 mL Falcon tube. The tube was incubated at 37°C and 250 rpm overnight to prepare the starting culture. For all characterisation assays, 1% (v/v) of the starting culture was transferred into 10 mL of fresh LB broth containing the relevant antibiotics. Cultivations in 96 deep‐well plates were performed in 300 μL LB broth under the same conditions as those used for the Falcon tube cultures. For the cultivation of AlkS mutant library transformants, M9 medium was employed, consisting of 1 × M9 salts (Gibco, Grand Island, NY, USA), 2 mM MgSO_4_, 0.1 mM CaCl_2_.

Ampicillin (Amp^R^) stock solution was prepared by dissolving ampicillin sodium salt in Milli‐Q water to achieve a final concentration of 100 mg/mL (1000× working concentration). Chloramphenicol (Cm^R^) stock solution was made by dissolving chloramphenicol powder in dimethyl sulfoxide (DMSO) to a final concentration of 34 mg/mL (1000× working concentration). Both stock solutions were sterilised using a 0.2 μm filter.

### 2.2 Cloning

All plasmids used in this study are shown in Table S2, and DNA primers are listed in Table S3.

DNA fragments containing the gene of interest and the vector backbone were amplified using PrimeSTAR Max DNA Polymerase (Takara Bio, Kusatsu, Shiga, Japan) in a thermocycler (Thermo Fisher Scientific, Waltham, MA, USA). This was followed by template removal via digestion with *Dpn*I (New England Biolabs, Beverly, MA, USA) from the PCR products. All double‐stranded DNA (dsDNA) was purified using the QIAquick PCR Purification Kit (QIAGEN, Düsseldorf, Germany) before proceeding with the Gibson assembly process, conducted using the NEBuilder HiFi DNA Assembly Kit (New England Biolabs, Beverly, MA, USA). The assembled plasmids were then transformed into *E. coli* DH5α (New England Biolabs, Beverly, MA, USA) via heat shock. Plasmid extraction was performed using the QIAprep Spin Miniprep Kit (QIAGEN, Düsseldorf, Germany).

The size of the dsDNA was confirmed through nucleic acid electrophoresis. For this, 1% agarose was dissolved in 1 × TAE buffer and 0.01% (v/v) SYBR Safe DNA Gel Stain (Invitrogen, Carlsbad, CA, USA) was added after the mixture cooled to about 60°C. The dsDNA samples, mixed with 6 × TriTrack DNA Loading Dye (Thermo Fisher Scientific, Waltham, MA, USA), were run alongside the GeneRuler 1 kb DNA Ladder (Thermo Fisher Scientific, Waltham, MA, USA) as a molecular size marker. Electrophoresis was conducted at 130 V for 20 min using the PowerPac Basic Power Supply (Bio‐Rad, Hercules, CA, USA) to separate the DNA fragments. The dsDNA concentrations were measured using NanoDrop Microvolume Spectrophotometers (Thermo Fisher Scientific, Waltham, MA, USA). Sanger sequencing to verify the target DNA sequences was performed by Source Bioscience (Nottingham, UK). All procedures adhered to the recommendations of suppliers.

### 2.3 Electroporation

To prepare electrocompetent *E. coli* BW25113 cells, a single colony was initially inoculated into a 50 mL Falcon tube containing LB broth and incubated overnight to establish a starting culture. The following day, 200 μL of this culture was used to inoculate fresh LB broth and grown until the cells reached the mid‐exponential growth phase, as indicated by an OD_600_ of 0.4–0.6. The broth was then transferred to 2 mL Eppendorf tubes and centrifuged at 6000 rpm for 5 min to pellet the cells at 4°C. The supernatant was discarded, and the cells were resuspended in 1 mL of chilled 10% (v/v) glycerol. This washing step was repeated three times to thoroughly remove residual media. After the final centrifugation, the supernatant was removed, and the cell pellet was resuspended in approximately 100 μL 10% (v/v) glycerol, preparing the cells for freezing or immediate use in electroporation.

For the electroporation of *E. coli* BW25113 with the desired plasmid, electrocompetent cells were first thawed on ice. 1–3 μL plasmid DNA (around 100 ng) was then added to the cells. The electrocompetent cells and DNA were gently mixed, and the mixture was allowed to sit on ice for 10 min. The mixture was then transferred to a chilled electroporation cuvette, and a pulse was delivered using a micropulser (Bio‐Rad, Hercules, CA, USA) set at 1.8 kV. After electroporation, the mixture was allowed to rest on ice for an additional 5 min, then 950 μL SOC (Super Optimal broth with Catabolite repression) medium (Thermo Fisher Scientific, Waltham, MA, USA) was added to facilitate cell recovery. The cells were incubated in a shaker at 37°C and 250 rpm for 1 h. Finally, the cells were plated on an LB agar plate containing the appropriate antibiotic and incubated overnight at 37°C.

### 2.4 AlkS Mutant Library Generation, Screening, and Analysis

AlkS variants were generated using epPCR with the GeneMorph II Random Mutagenesis Kit (Agilent, Santa Clara, California, USA). The template fragment used excluded both the start codon and a putative C‐terminal DNA binding domain (residues 839–858). The resulting *alkS* plasmid library was transformed into *E. coli* BW25113.

For FACS‐based screening, transformants harbouring the *alkS* library were initially recovered in SOC medium for 1 h, then transferred into 10 mL of LB medium supplemented with 34 μg/mL Cm^R^ and 100 μg/mL Amp^R^ and incubated for an additional 2 h at 37°C. After this, the cells were centrifuged for 10 min at 4°C and 3500 rcf, resuspended in 10 mL of M9 medium containing 0.2 g/L glucose, and incubated overnight. The following day, the culture was diluted tenfold in M9 medium containing 4 g/L glucose and incubated for 3 h before being induced with 10 mM isobutanol for another 3 h. The cells were then centrifuged, washed three times with phosphate‐buffered saline (PBS, Thermo Fisher Scientific, Waltham, MA, USA), and resuspended in 1 mL of PBS to adjust the OD_600_ to approximately 0.02 for FACS analysis.

Four rounds of FACS were conducted using a BD FACSAria III Cell Sorter (BD Biosciences, San Jose, CA, USA). Gating was set based on side scatter height (SSC‐H) and forward scatter height (FSC‐H) to exclude cell debris and potential microbial contaminants. The populations exhibiting the highest GFP values were sorted and cultured in LB broth containing 34 μg/mL Cm^R^ and 100 μg/mL Amp^R^ at 37°C and 250 rpm for 2 h. Subsequently, 1% of these strains were inoculated into M9 medium supplemented with 0.2 g/L glucose and incubated overnight. For single cell colony collection, the final round cells were sorted onto square LB agar plates supplemented with the same antibiotics and incubated at 37°C overnight. Single colonies were then picked into 96 deep‐well plates containing 300 μL of fresh LB broth with the antibiotics and incubated overnight for further fluorescence assay.

### 2.5 Fluorescence Assay

After each mutation, the response of the biosensor for isobutanol and other alcohols, including methanol, ethanol, n‐propanol, isopropanol, n‐butanol, tert‐butanol, n‐pentanol, and isopentanol, was assessed. Initially, 1% of an overnight starting culture was inoculated into a 10 mL Falcon tube containing 1 mL of fresh LB broth supplemented with 34 μg/mL Cm^R^ and 100 μg/mL Amp^R^ and incubated at 37°C, 250 rpm, for 2 h. A specified concentration of the relevant alcohol was then added, and the culture was incubated for an additional 8 h. Next, the cells were centrifuged, washed with PBS, and finally, 100 μL of the culture broth was transferred to a 96‐well optical‐bottom plate, and sfGFP intensity was measured (excitation at 485 nm, emission at 520 nm, and absorbance measurement under 600 nm) using a Biotek Cytation 3 imaging plate reader (Agilent, CA, USA).

### 2.6 AlkS Structure Modelling and Molecular Docking

Protein structure and functional sites of AlkS were retrieved from UniProt (https://www.uniprot.org/uniprotkb/P17051/entry). Tertiary structure predictions for AlkS mutants were performed using AlphaFold 3 (Abramson et al. 2024). The 3D structures of both AlkS and its mutants were visualised and analysed using PyMOL v3.0. Molecular structures of n‐pentanol and isobutanol were obtained from PubChem (https://pubchem.ncbi.nlm.nih.gov/compound/6276). For visualisation of binding residues, Discovery Studio v16.1.0 was employed. Molecular docking of n‐pentanol and isobutanol with AlkS and its mutants was conducted using AutoDock Vina. Residues for targeted mutagenesis were selected based on docking proximity (< 4 Å from ligand).

### 2.7 Statistical Analysis

Each experiment was conducted with biological replicates to ensure reliability. Hill curves were fitted using a non‐linear regression model as asymmetrical (five‐parameter) logistic dose–response curves (Prism 10.1.2, GraphPad, San Diego, CA, USA). Statistical significance was determined using an unpaired t‐test. Interpretation of results was as follows: a *p*‐value greater than 0.05 indicated no significant difference (ns); a *p*‐value between 0.01 and 0.05 was considered to show low significance (*); a *p*‐value between 0.001 and 0.01 denoted medium significance (**); and a *p*‐value of 0.001 or less indicated high significance (***).

## 3 Results and Discussion

### 3.1 AlkS Mutant Library Generation, Screening, and Analysis

The rational engineering of the AlkS‐PalkB biosensor is described in detail in Supporting Section S1 and S2. Briefly, site‐directed mutagenesis and computational modelling were employed to shift the ligand specificity of AlkS from n‐pentanol toward isobutanol, and a dual‐plasmid system was implemented to improve the biosensor's dynamic range. However, rational design alone was insufficient to confer specificity toward isobutanol successfully.

Therefore, error‐prone PCR (epPCR) was employed to generate a library of *alkS* mutants. The wild‐type AlkS protein, comprising 838 amino acids, was annotated in UniProt, with its DNA‐binding domain located from the 839th to the 858th amino acid. To avoid mutations in the start codon or the DNA‐binding domain, the epPCR template was specifically designed to include amino acids 2nd through 838th. The scale of the resulting mutant library was determined by plating transformants containing 1 μL *alkS* variants within the DH5α strain, yielding approximately 2.8 × 10^5^ variants. The mutation rate within the library was subsequently assessed, revealing an average of 5.1 amino acid substitutions per AlkS variant (excluding silent mutations), 7.8 base substitutions per gene, and a silent mutation rate of 29%.

The screening of *E. coli* BW25113 transformants harbouring the AlkS variants library was conducted across four rounds of fluorescence‐activated cell sorting (FACS). Initially, to eliminate variants exhibiting high basal fluorescence, the first round of FACS selectively isolated cells from the population displaying the lowest fluorescence signal (38%) without isobutanol induction. In subsequent rounds, cells were sorted from populations showing the highest fluorescence response to 10 mM isobutanol induction. Between each round, cells were regrown and re‐induced with 10 mM isobutanol to ensure continuous enrichment of populations with the desired traits. During each FACS round, gates were set based on side scatter height (SSC‐H) and forward scatter height (FSC‐H) to accurately isolate the *E. coli* cells demonstrating the most promising responses. This iterative screening process, coupled with strategic gating during FACS, proved crucial in refining the library to isolate mutants that responded more robustly to isobutanol and reduced background noise from non‐specific fluorescence (Figure S3).

**FIGURE 2:**
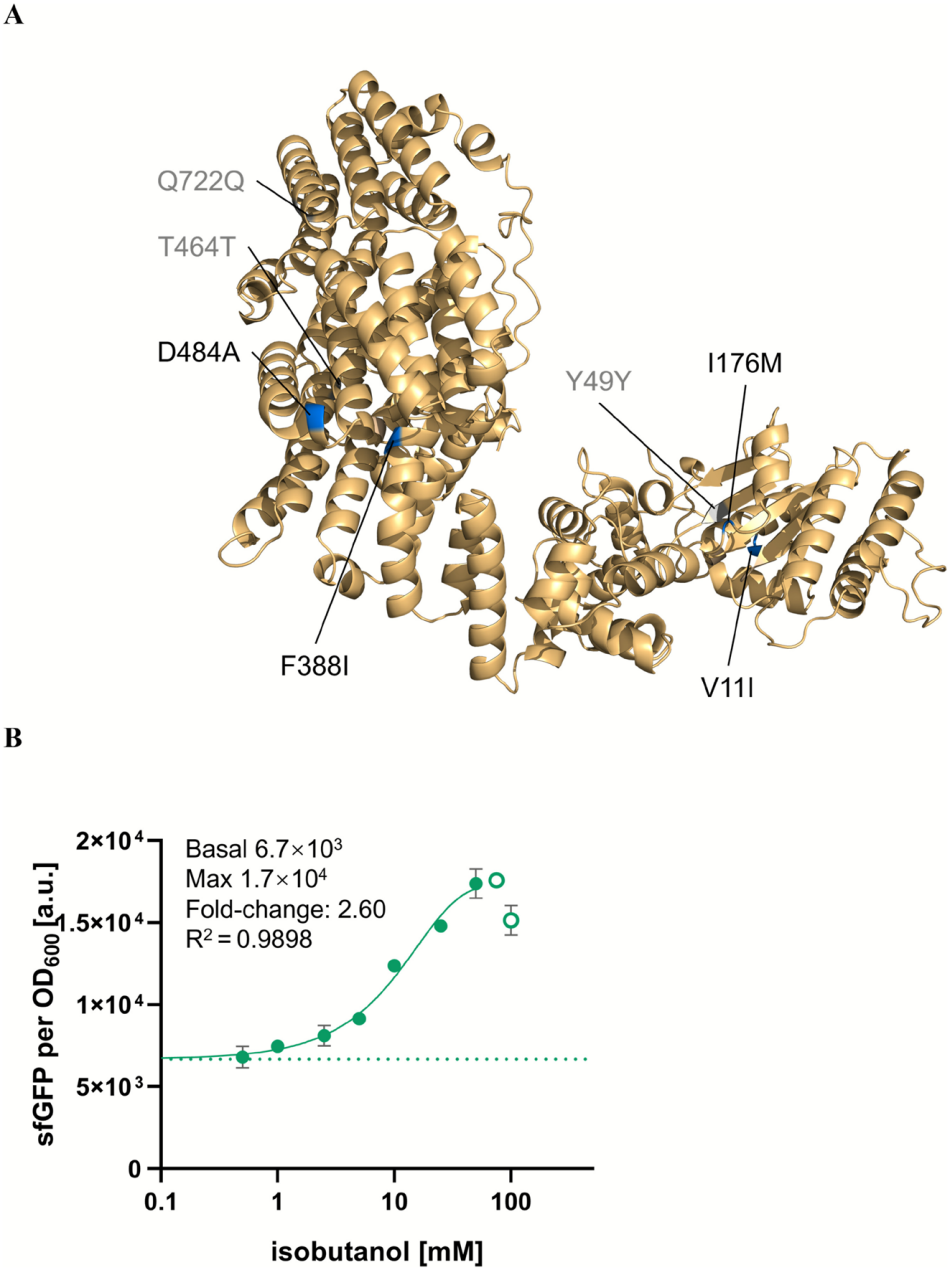
(A) Homology model of AlkS with labelled mutation sites. Synonymous mutations are shown in grey, while non‐synonymous mutations are shown in blue. (B) Dose–response curve of *E. coli* BW25113‐*alkS_mut‐P*_*alkB*_ (carries *pACYC184‐P*_*alkS*_ *‐alkS* (*mut*) and *pUC19‐P*_*alkB*_ *‐sfgfp* plasmids) biosensor to different concentrations of isobutanol from 0 to 100 mM. The empty circles were excluded due to strong growth impediment. Dashed lines represent sfGFP fluorescence in the absence of exogenously supplied isobutanol. The experiments were carried out in triplicate with error bars representing the standard deviation from the mean.

After four rounds of FACS, 117 colonies were viable on the LB agar plates. Among these, colonies exhibiting the highest fluorescence signals were selected for further characterisation. Sequencing revealed that all the fluorescent colonies shared the same eight mutations (V11I, Y49Y, I176M, F388I, F424L, T464T, D484A, and Q722Q) (Figure 2A). The dose–response curve of the highest fluorescence expression mutant (Figure 2B) showed a 2.60‐fold increase in response to isobutanol. Notably, isobutanol concentrations of 100 mM or higher were found to cause growth arrest in *E. coli* , potentially leading to deviations in fluorescence measurements. These deviations could arise from a lower OD_600_ readout rather than an actual increase in sfGFP production. It is hypothesized that isobutanol impacts the cell membrane composition and disrupts intracellular environment homeostasis, which might reduce the cell growth rate. However, transcription and translation rates could still remain high, allowing sfGFP to accumulate and leading to elevated fluorescence levels at higher isobutanol concentrations. Consequently, when a larger sfGFP signal is divided by a smaller OD_600_, the resultant fluorescence readout per OD_600_ is inflated. The fluorescence data at isobutanol concentrations of 75 mM and 100 mM were thus considered inaccurate. Due to these observations, the operational range for this biosensor candidate was established between ~1 and 50 mM isobutanol to avoid the confounding effects of isobutanol toxicity. The high basal expression level of the biosensor likely resulted from the high expression level of the reporter module.

**FIGURE 3:**
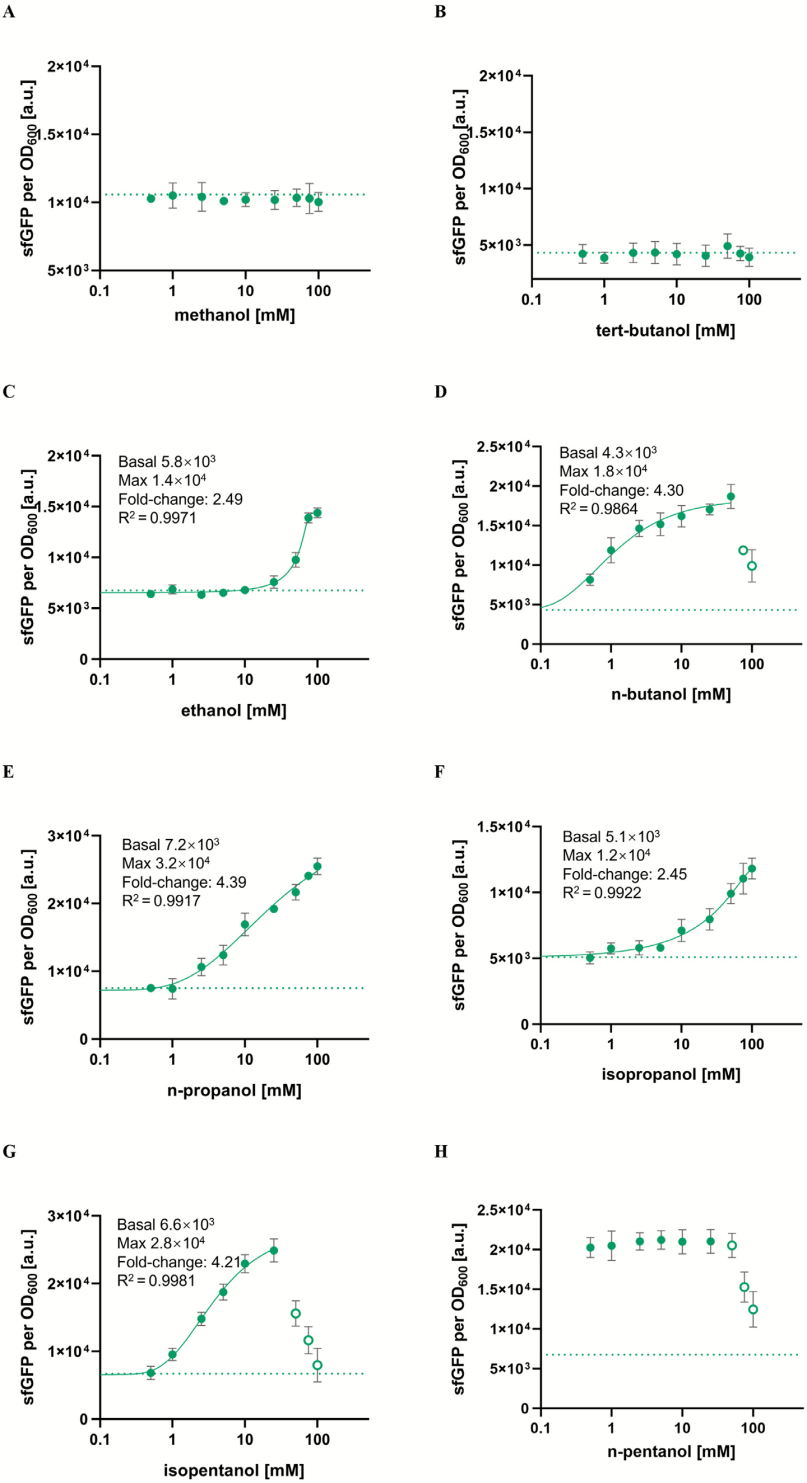
Dose–response curves of *E. coli* BW25113‐*alkS_mut‐PalkB* (carries *pACYC184‐PalkS‐alkS* (*mut*) and *pUC19‐PalkB‐sfgfp* plasmids) biosensor with (A) methanol; (B) tert‐butanol; (C) ethanol; (D) n‐butanol; (E) n‐propanol; (F) isopropanol; (E) n‐butanol; (G) isopentanol; (H) n‐pentanol. The empty circles were excluded due to strong growth impediment. Dashed lines represent sfGFP fluorescence in the absence of exogenously supplied isobutanol. The experiments were carried out in triplicate with error bars representing the standard deviation from the mean.

Experiments were conducted to evaluate the specificity of the variant isolated through fluorescence‐activated cell sorting (FACS), named AlkS_mut, for binding to C1–C5 short‐chain alcohols. The mutant biosensor produced the highest fluorescence signal in response to n‐pentanol, similar to the wild‐type, although the signal plateaued rapidly, indicating limited sensitivity to concentration changes. In contrast, the AlkS_mut displayed inducibility by isobutanol, marking a distinct functional divergence from the wild‐type. This variant also showed enhanced response for several C2–C5 alcohols, suggesting modifications in its binding pocket. However, neither methanol (Figure 3A) nor tert‐butanol (Figure 3B) effectively activated the AlkS_mut, likely due to structural incompatibilities. For ethanol (Figure 3C), the mutant exhibited a narrow response range (~25–75 mM). This characteristic is particularly advantageous, as ethanol is a common byproduct of microbial iobutanol fermentation processes, and the lack of response at low ethanol concentrations reduces interference with green fluorescence intensity, improving the biosensor's selectivity in common fermentation environments. Interestingly, the AlkS_mut exhibited a substantial fluorescence response at relatively low concentrations of n‐butanol (approximately 2.5 mM; Figure 3D). Although the signal continued to rise at higher concentrations, up to ~50 mM, the subsequent increases were modest and, when accounting for the error bars, may not be statistically significant. This gradual signal increase at higher concentrations poses a challenge for accurate quantification in applications involving n‐butanol. On the other hand, the variant demonstrated significant potential for detecting n‐propanol (Figure 3E), isopropanol (Figure 3F), and potentially isopentanol (up to ~25 mM) (Figure 3G), suggesting expanded utility for these alcohols. For n‐pentanol (Figure 3H), however, the response range narrowed substantially, with maximum green fluorescence occurring at minimal concentrations, making the mutant unsuitable for n‐pentanol detection.

These observed changes in specificity suggest structural modifications within the AlkS_mut binding pocket, now more effectively accommodating alcohols with shorter backbones or side chains. This behaviour aligns with the hypothesis that transcription factors (TFs) typically exhibit an initial broadened selectivity, which gradually evolves into high specificity for distinct ligands. To transcend this promiscuity, careful and incremental evolutionary steps are necessary, meticulously balancing specificity with thermodynamic stability (Galvão et al. 2007). The AlkS_mut obtained from FACS shows broadened response for various short‐chain alcohols and reduced response for its natural ligand. This behaviour is consistent with the proposed model of TF evolution. To further refine this variant into a TF with high specificity for isobutanol, continuous evolution may be required. This could be achieved through additional rounds of directed evolution or further rational design interventions, each aimed at enhancing its specificity. Such efforts would not only fine‐tune the binding characteristics but also optimise the biosensor performance for practical applications.

### 3.2 Optimisation of Biosensor Function

Despite the improvements achieved through fluorescence‐activated cell sorting, which enhanced the fold‐change of an AlkS biosensor variant from 1.81 to 2.60, the dynamic range of the biosensor remains suboptimal for high‐throughput screening applications. To address this limitation and extend the biosensor applicability, two primary strategies were implemented for optimisation: structure‐guided site‐directed mutagenesis and promoter modification of PalkB (Bahls et al. 2022; Wu et al. 2022). These approaches are designed to enhance the biosensor sensitivity by modulating protein interactions and transcriptional efficiency.

Sequencing of the AlkS variant revealed five mutations (V11I, I176M, F388I, F424L, and D484A) and three silent mutations (Q722Q, T464T, Y49Y). The mutations were located away from the DNA‐binding domain and the predicted ligand‐binding pocket. This distribution supports the hypothesis that modifications to the effector binding pocket indirectly influence the overall function of the transcription factor. It underscores the pivotal role of the broader protein context in determining whether binding induces the necessary conformational changes for transcriptional activation. This insight also explains why mutations distant from the binding pocket or DNA‐binding site can still significantly affect the selectivity of AlkS, highlighting the broader protein context impact on binding dynamics.

**FIGURE 4:**
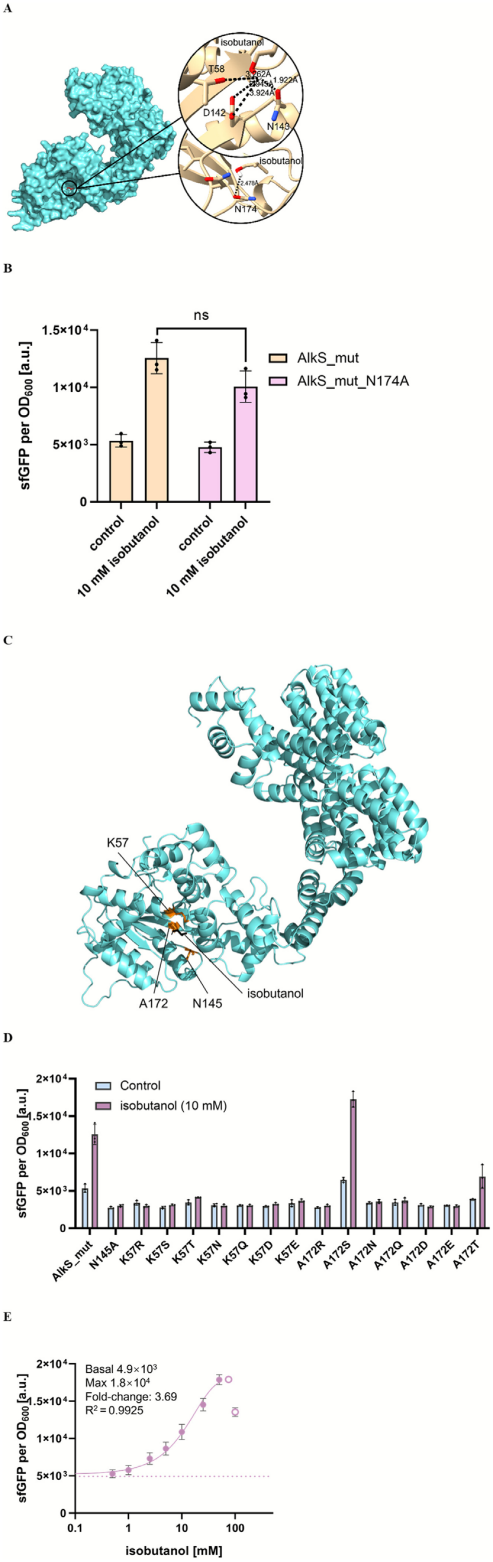
(A) Schematic diagram of docking simulations between AlkS mutant and isobutanol. AlkS is displayed in light blue, isobutanol is highlighted in red. Predicted binding pocket and potential key residues (T58, D142, N143 and N174) that could form H‐bonds with isobutanol are demonstrated in black circles. (B) Alanine scanning results for predicted key residues (N174) during the binding between AlkS mutant and isobutanol. (C) Response of different variants of single‐point AlkS mutant to 10 mM isobutanol. (D) Rationally designed sites for site‐directed mutagenesis. Protein is coloured cyan, isobutanol in black, and mutation sites in orange. (E) Dose–response curves of *E. coli* BW25113‐*alkS_mut_A172S‐PalkB*, carrying *pACYC184‐PalkS‐alkS* (*mut*)*‐A172S* and *pUC19‐PalkB‐sfgfp* plasmids with different concentrations of isobutanol from 0 to 100 mM. The empty circles were excluded due to strong growth impediment. Dashed lines represent sfGFP fluorescence in the absence of exogenously supplied isobutanol. The experiments were carried out in triplicate with error bars representing the standard deviation from the mean.

In the absence of current 3D structural data for the AlkS mutant, its spatial configuration was predicted using AlphaFold 3. Molecular docking, performed with AutoDock Vina, was utilised to define the binding pocket of the AlkS mutant for isobutanol. Analysis of free energy changes and hydrogen bond formation potential indicated an optimal binding configuration, illustrated in Figure 4A. Docking results suggested that the binding pocket configuration of the AlkS mutant for isobutanol closely resembles that of the wild‐type AlkS for n‐pentanol. Notably, asparagine at position 174 (N174) emerged as a potential novel residue involved in the binding mechanism, possibly acting as a hydrogen bond receptor for isobutanol. To confirm the role of N174, it was substituted with alanine, and the impact on fluorescence output was evaluated, as depicted in Figure 4B. This substitution resulted in a slight decrease in fluorescence output, suggesting that N174 plays a modest yet significant role in the binding dynamics of the AlkS mutant with isobutanol.

Given the observed similarities in the binding pocket configurations between the AlkS mutant with isobutanol and the wild‐type AlkS with n‐pentanol, we adopted a similar site‐directed mutagenesis approach aimed at minimising steric hindrance and maximising the potential for effective hydrogen bond formation. This includes the mutation of Asn145 to Ala, which has a shorter side chain, and the mutations of Lys57 and Ala172 to residues such as Arg, Ser, Thr, Asn, Gln, and Glu, whose side chains may form additional hydrogen bonds with alcohols (Figure 4C).

As shown in Figure 4D, the mutation A172S in the AlkS variant demonstrated an increased sensitivity specifically for isobutanol, distinguishing it from other mutants, which did not show inducibility by isobutanol. This particular mutation, located strategically near the binding site, appears to modify the pocket in a manner that enhances compatibility with isobutanol molecular structure, potentially by improving the alignment of hydrogen bonding sites or reducing steric hindrances that reduce effective binding.

Further quantitative evaluation of the biosensor with A172S variant revealed a notable increase in its responsiveness to isobutanol. The fold‐change in fluorescence response improved from 2.60 to 3.69, representing a 42% enhancement in dynamic range, as detailed in Figure 4D. However, despite these promising results, the operational range for isobutanol detection remained restricted to 0–50 mM, primarily due to the cytotoxic effects of higher isobutanol concentrations on the host cells. This limitation underscores the delicate balance between biosensor sensitivity and the physiological tolerance of the biosensor host. This analysis highlights the effectiveness of site‐directed mutagenesis in refining biosensor performance and suggests its broad‐spectrum utility for enhancing biosensor capabilities across various applications. Therefore, moving forward, a detailed examination of additional mutation sites in the AlkS variant is essential. Such an analysis should be holistic, considering the dynamics of the entire protein to ensure that mutations contribute positively to the biosensor functionality without destabilising the protein or diminishing its overall efficiency.

The core promoter region, particularly the −35 and −10 region, is fundamental to the binding process of RNA polymerase (RNAP), and it significantly impacts the strength of the target promoter. Observations indicate that core promoter sequences often show species‐specific biases, which present considerable opportunities for modifying promoter strength in various organisms (Myers et al. 2021). Though the detailed activation mechanism of the PalkB promoter remains unclear, it has been reported that PalkB activation in *Pseudomonas putida* is independent of *σ* ^S^ (Canosa et al. 1999). In *E. coli* , the promoter is likely recognised by σ^70^ instead of σ^38^ through its interaction with the −10 and −35 elements. In the presence of the inducer, AlkS binds to the inverted repeat sequence upstream of the −35 element of the PalkB promoter, potentially promoting its interaction with RNA polymerase (RNAP) to activate transcription (Canosa et al. 2000). Previous studies have shown that variations in the −35 and −10 elements can significantly influence transcriptional efficiency. For example, replacement of the *ompC* wild‐type −35 region with the consensus sequence markedly enhanced OmpR‐induced promoter activity (Tsung et al. 1990). Therefore, a promoter sequence that more closely matches the *E. coli* −35 consensus is expected to facilitate RNAP recruitment and potentially strengthen the interaction between the inducer and RNAP.

In this study, substitution of the PalkB −10 element with the *E. coli* consensus sequence resulted in a substantial increase in basal expression, as indicated by the visibly intense green coloration of *E. coli* cultures (data not shown). Consequently, this variant was excluded from further analysis. It has also been reported that alterations in the −10 sequence can modulate the interaction between inducers and RNAP, thereby enabling transcriptional activators to function as both positive and negative regulators of gene expression (Tsung et al. 1990).

**FIGURE 5:**
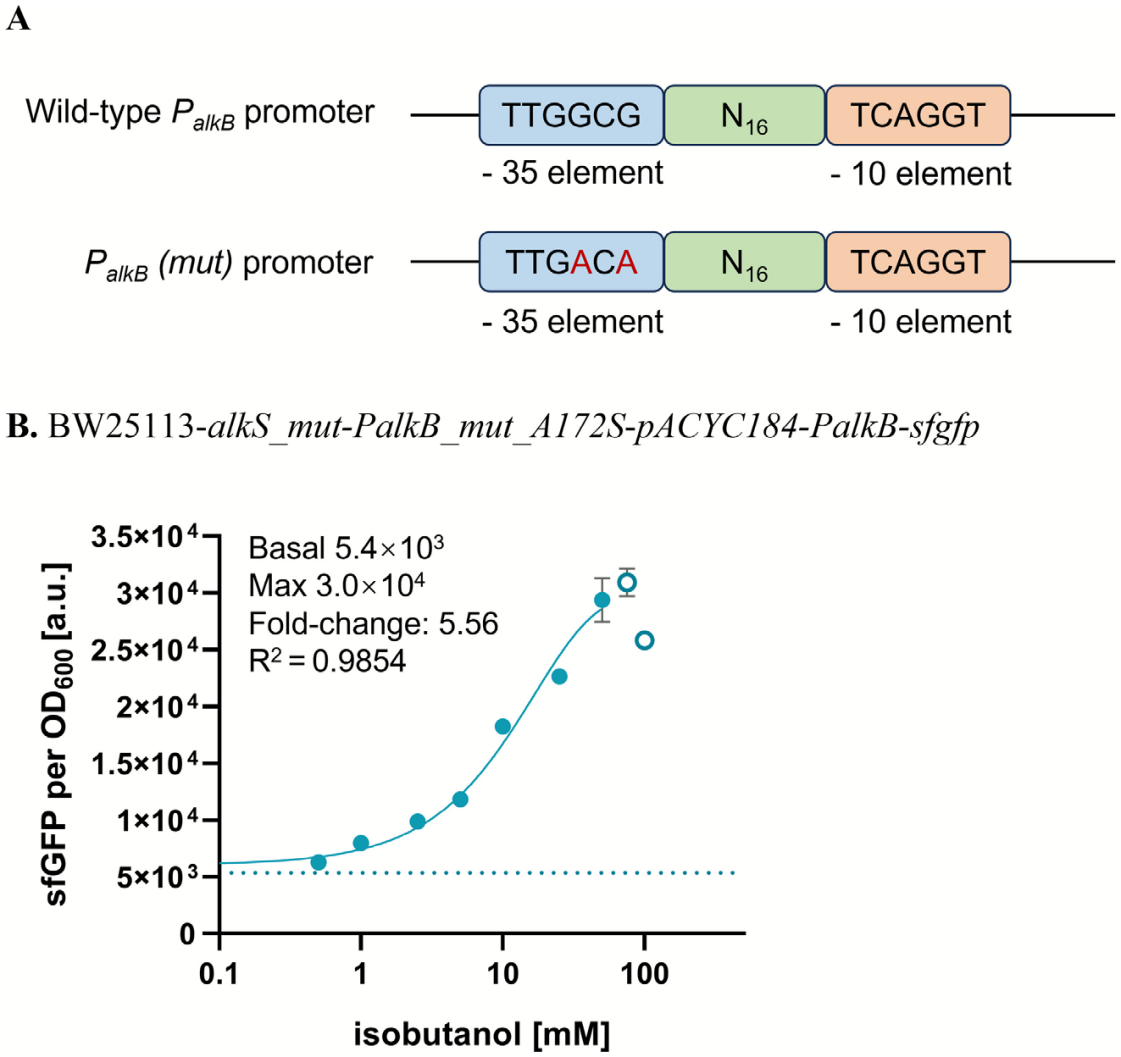
(A) Schematic diagram for construction of the *alkB* promoter and its variant. The 16‐bp spacer sequence between the −35 and −10 regions are shown as N16, the mutations within −35 are highlighted in red. (B) Dose–response curves of *E. coli* BW25113‐*alkS_mut_A172S‐PalkB_mut*, carrying *pACYC184‐PalkS‐alkS* (*mut*)‐*A172S* and *pUC19‐PalkB* (*mut*)‐*sfgfp* plasmids with different concentrations of isobutanol from 0 to 100 mM. Dashed lines represent sfGFP fluorescence in the absence of exogenously supplied isobutanol. The empty circles were excluded due to strong growth impediment. The experiments were carried out in triplicate with error bars representing the standard deviation from the mean.

Thus, to improve the binding of RNAP to *PalkB*, the −35 region of PalkB was modified to align with the consensus sequence of *E. coli* (TTGACA) (De Mey et al. 2007). However, modifications to the −10 region resulted in substantial leaky expression of the biosensor. Consequently, only the −35 region mutant was further evaluated for its performance (Figure 5A). In the presence of the A172S mutation, the fold‐change increased from 3.69 (Figure 4E) to 5.56 (Figure 5B), representing an enhancement of about 51% due to the promoter mutation. Overall, in comparison to the initial construct without the A172S substitution, the fold‐change increased from 2.60 (Figure 2B) to 5.56 using the optimised construct with the A172S substitution and the *PalkB_mut* promoter (Figure 5B), corresponding to an approximate 114% improvement.

The operational window of the engineered biosensor (~1–50 mM, ≈0–3.7 g·L^−1^) aligns well with the concentration range typically observed in batch cultures used for HTS. For example, the yield of *Bacillus subtilis* is about 2.62 g/L in shake flasks (Li et al. 2011). In most laboratory‐scale studies, product titres usually remain below 5 g/L, and screening campaigns focusing on enzyme variant libraries or early strain engineering cycles generally operate well within this range (Shu et al. 2022; Xie et al. 2025). Under these conditions, the present biosensor provides a sensitive and convenient tool for guiding enzyme and strain improvement, and its use could be readily combined with strategies such as adaptive laboratory evolution and continuous perturbation tools (Moon et al. 2025; Xiao et al. 2025) to accelerate the identification of superior producers.

**FIGURE 6:**
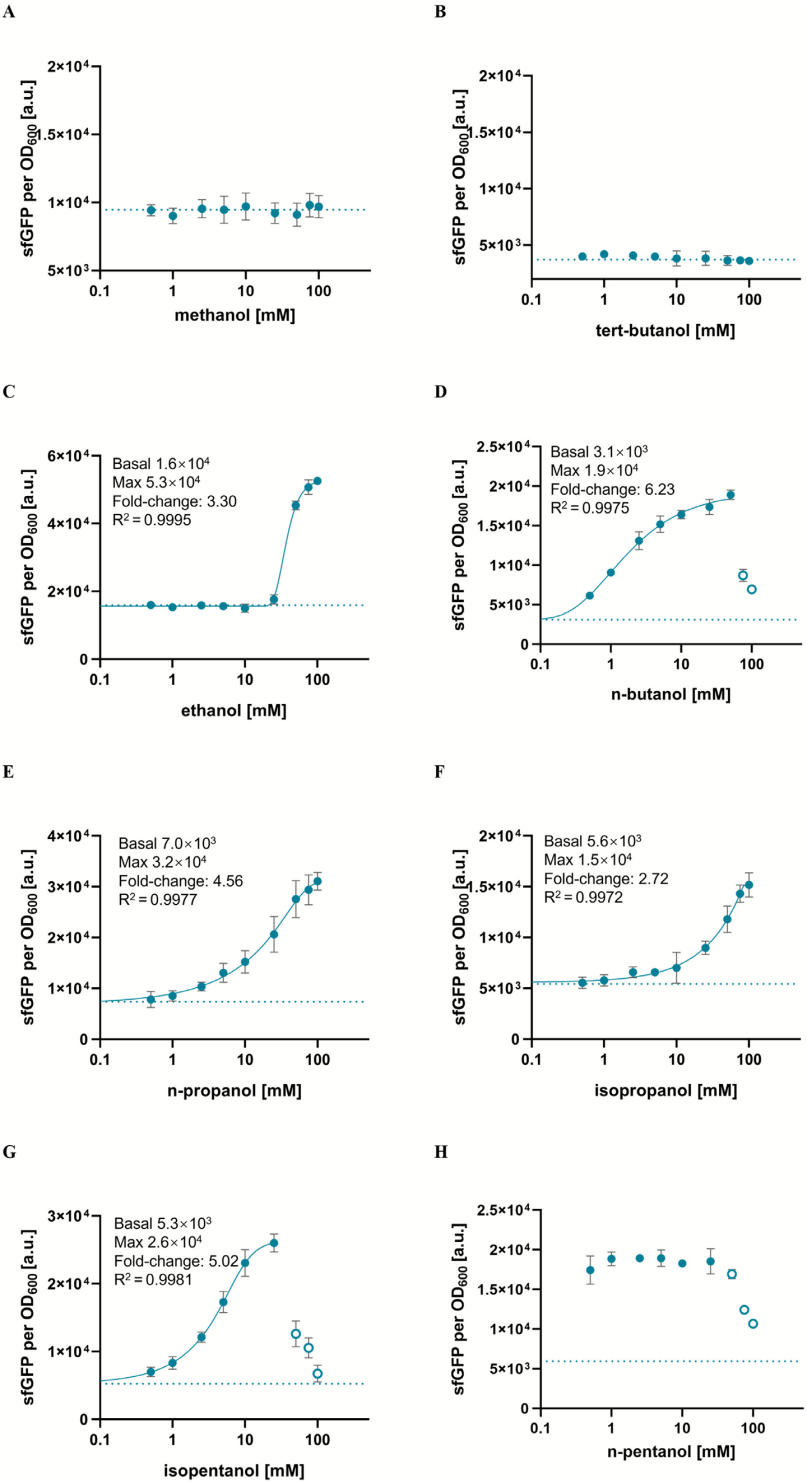
Dose–response curves of *E. coli* BW25113‐*alkS_mut_A172S‐PalkB_mut*, carrying *pACYC184‐PalkS‐alkS* (*mut*)‐*A172S* and *pUC19‐PalkB* (*mut*)‐*sfGFP* plasmids biosensor with (A) methanol; (B) tert‐butanol; (C) ethanol; (D) n‐butanol; (E) n‐propanol; (F) isopropanol; (G) isopentanol; (H) n‐pentanol. The empty circles were excluded due to strong growth impediment. Dashed lines represent sfGFP fluorescence in the absence of exogenously supplied isobutanol. The experiments were carried out in triplicate with error bars representing the standard deviation from the mean.

In addition to isobutanol, the specificity of the optimised AlkS biosensor was systematically evaluated with other C1–C5 short‐chain alcohols (Figure 6). The optimisation process, involving point mutations and promoter engineering, was specifically designed to expand the dynamic range and fold change in response to target ligands. Consistent with these design principles, the experimental results confirmed that the optimised AlkS variant remained unresponsive to methanol and tert‐butanol. This lack of activation underscores the structural incompatibilities between these alcohols and the modified binding pocket of the biosensor, aligning with previous observations of their non‐reactivity. Notably, the optimised biosensor exhibited the most pronounced improvement in dynamic range and fold change toward isobutanol, compared to other alcohols tested. Modest increases were also observed for n‐butanol, n‐propanol, isopropanol, and isopentanol, but the response toward isobutanol was substantially larger (Table 1). This increased sensitivity illustrates structural refinements in the binding pocket that strengthen interactions with specific ligands. Moreover, the optimised AlkS variant is well suited for detecting these short‐chain alcohols, which arise from distinct biosynthetic routes, thereby minimising potential cross‐interference (Lan and Liao 2013; Runguphan et al. 2021; Walther and François 2016). The enhanced performance of the AlkS variant, characterised by an improved fold change, is especially advantageous for HTS applications.

**TABLE 1:** Summary of key characteristics and improvements of the optimised biosensor compared to the initial biosensor (AlkS_mut) obtained from FACS screening.

Alcohol	Operating range [mM]	Fold change (initial/optimised)	Increase [%]
Isobutanol	~1–50	2.60/5.56	114
n‐butanol	~0.5–50	4.30/6.23	45
Ethanol	~25–75	2.49/3.30	33
n‐propanol	~5–75	4.39/4.56	4
Isopropanol	~5–75	2.45/2.72	11
Isopentanol	~0.5–25	4.21/5.02	19

This substantial enhancement underscores the potential of tailored promoter engineering combined with targeted protein modifications to significantly improve the sensitivity and specificity of biosensors. Such advancements refine detection capabilities and broaden the applicability of biosensors across various biotechnological applications, enabling more precise control over biological processes in different host organisms.

Despite these notable improvements, several limitations persist, such as the high basal GFP expression level of the biosensor and the cytotoxic effects of isobutanol on *E. coli* cells. The basal expression is typically attributed to the basal level transcription of the reporter gene, which could be mitigated by incorporating degradation tags or degrons into the target proteins (Cameron and Collins 2014). For instance, employing the tobacco etch virus protease (TEV) and the protein degradation tag Ssr to control the degradation of GFP and the TF has proven effective. In the absence of an inducer, both the TF and GFP are degraded, reducing unnecessary expression. Conversely, the presence of an inducer activates TEV expression, which leads to the cleavage of the degradation tag and the subsequent generation of an output signal (Zhang et al. 2022).

Furthermore, enhancing the dynamic range and managing leakiness of the biosensor could be achieved through various strategies targeting the inducible promoter. These strategies include altering the number and position of operator sites, modifying the spacer sequence between the −35 and −10 regions, and randomising core promoter regions to better fit the host transcriptional machinery (Chen et al. 2019; Murphy et al. 2007). Additionally, developing isobutanol‐tolerant strains or employing a cell‐free system based on existing gene circuits could address issues inherent to cell‐based systems, potentially expanding the operating range of the biosensor. A cell‐free system, in particular, might offer a viable alternative for applications where cell viability is a concern, providing a controlled environment for the biosensor components without the complexities of cellular metabolism (Sherkhanov et al. 2020).

These improvements and proposed strategies highlight the iterative nature of biosensor development, where each cycle of design, build, test, and learn not only enhances the current system but also provides insights into new avenues for further refinement. The combination of genetic engineering tools and synthetic biology principles continues to push the boundaries of what is possible in the field of biosensor technology, paving the way for more robust, efficient, and versatile diagnostic and monitoring tools in biotechnology and beyond.

## 4 Conclusions

In conclusion, we have significantly improved the dynamic range and specificity of the isobutanol biosensor, demonstrating the power of synthetic biology in addressing key biotechnological challenges. While bioisobutanol has shown promise as a renewable fuel, its commercialisation has faced hurdles, including technological barriers in microbial fermentation, high production costs, and regulatory constraints. Our work contributes to overcoming these challenges by enabling more efficient strain screening. The integration of site‐directed mutagenesis and promoter engineering has led to a 114% increase in dynamic range, enhancing biosensor sensitivity and reducing cross‐reactivity with other short‐chain alcohols. These advancements pave the way for future biosensor development and support the broader goal of improving the economic and industrial viability of bioisobutanol production.

## Author Contributions

F.B., J.C., and X.W. conceived and designed the experiments. J.C., X.W., and C.Z. did the lab work. J.C. and X.W. did the literature review and prepared the manuscript. S.F., J.H., and F.B. helped to revise the manuscript. All authors read and approved the final manuscript.

## Funding

This work was supported by a Royal Society‐Newton Advanced Fellowship (NAF\R2\180721) and a UKRI–EU Underwrite UK Research and Innovation grant (EP/X032078/1).

## Conflicts of Interest

The authors declare no conflicts of interest.

## Supporting information


**Data S1:** mbt270288‐sup‐0001‐DataS1.docx.

## Data Availability

Data are available upon request to the corresponding author.
