# Supplementary material for: Engineering an AlkS‐PalkB Transcription Factor‐Based Biosensor With Improved Sensitivity to Isobutanol and Other Short‐Chain Alcohols
Source: Microb Biotechnol. 2025 Dec 25;18(12):e70288. doi: 10.1111/1751-7915.70288 (PMC12740625; doi:10.1111/1751-7915.70288)
Supplement: Supplementary file 1 — Data S1: mbt270288‐sup‐0001‐DataS1.docx. [file MBT2-18-e70288-s001.docx]

**Supplementary Material:**

S1 & S2. Rational Engineering of AlkS-PalkB Biosensor.

To improve the dynamic range of the AlkS-PalkB biosensor, a dual-plasmid system was implemented. In this design, the sensing module was carried on a low-copy-number plasmid, while the reporter module was placed on a high-copy-number plasmid, reducing metabolic burden and enhancing output signal (Figure S1A–B). Strains carrying both plasmids demonstrated an expanded dynamic range compared to the single-plasmid configuration, supporting the utility of this approach for biosensor optimization.

| **A**  **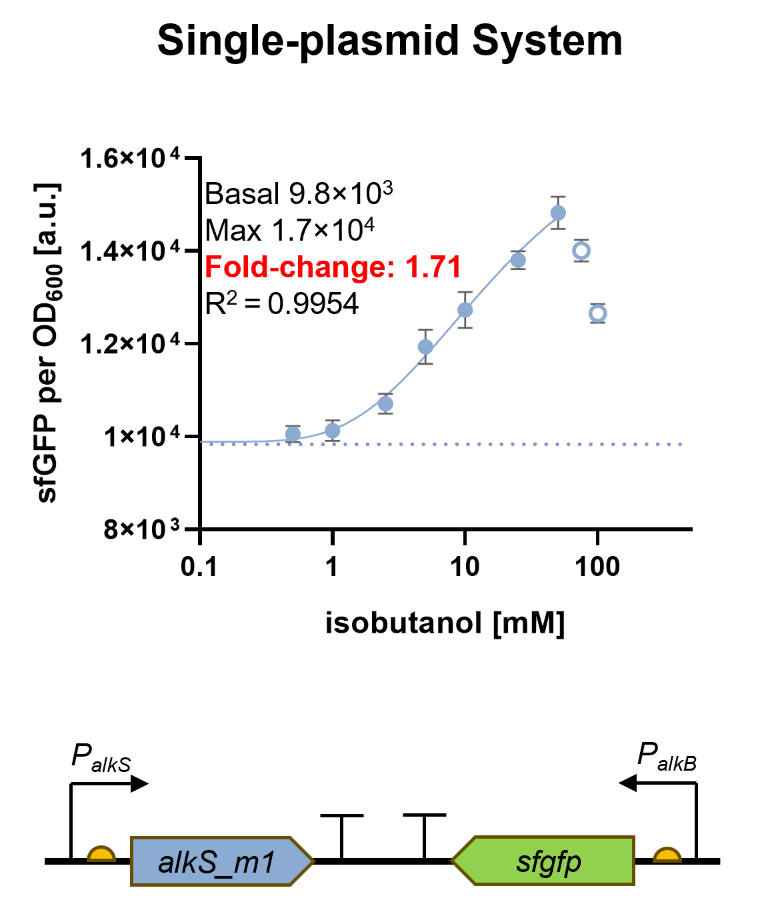** |
| --- |
| **B**  **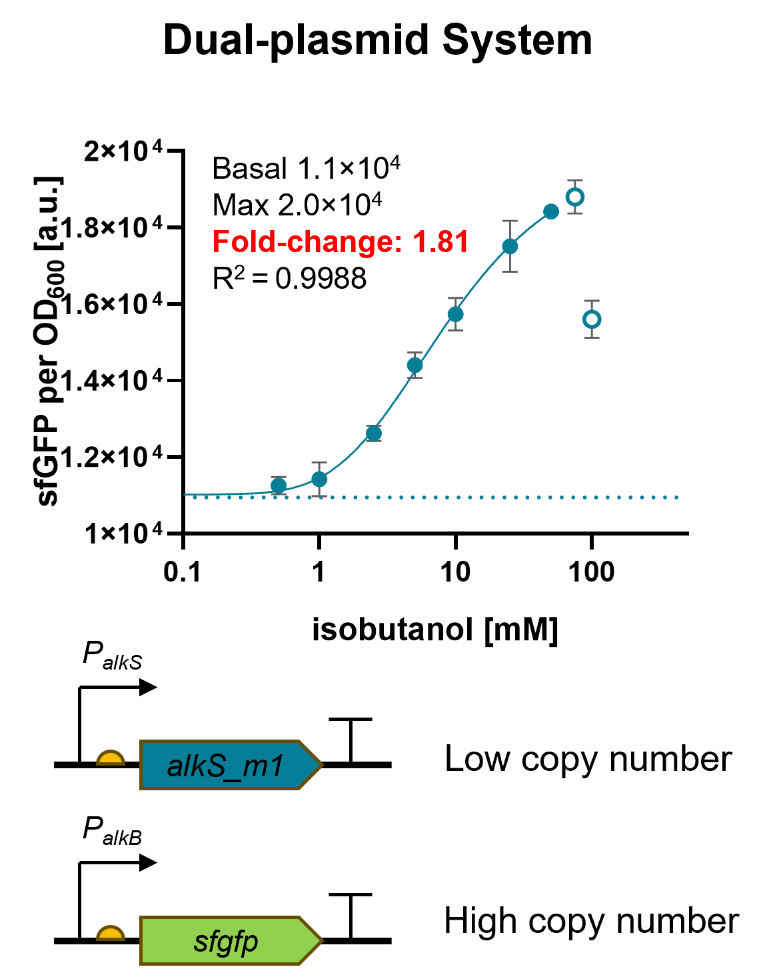** |
| Figure S1. Dose-response curves of (A) *E. coli* BW25113-*P_alkS_-alkS (D_e-p_)-P_alkB_-sfgfp*-1p biosensor and (B) *E. coli* BW25113-*P_alkS_-alkS (D_e-p_)*/*P_alkB_-sfgfp* biosensor. The empty circles were excluded due to strong growth impediment. The experiments were carried out in triplicate with error bars representing the standard deviation from the mean. |

To shift ligand specificity from n-pentanol toward isobutanol, computational docking simulations were first performed. The predicted structure of AlkS, generated by AlphaFold, was used in AutoDock Vina to identify the binding pocket and key residues potentially forming hydrogen bonds with n-pentanol (Figure S2A). Alanine scanning of these residues (T58, D142, and N143) confirmed their critical roles in ligand binding, as substitutions markedly reduced fluorescence in response to n-pentanol (Figure S2B).

Following this, site-directed mutagenesis of residues near the binding pocket was conducted to enhance interactions with isobutanol while reducing steric hindrance. Although certain mutations (e.g., A172S, N145A) improved responsiveness to n-pentanol, they did not confer substantial specificity toward isobutanol (Figure S2C).

| **A**  **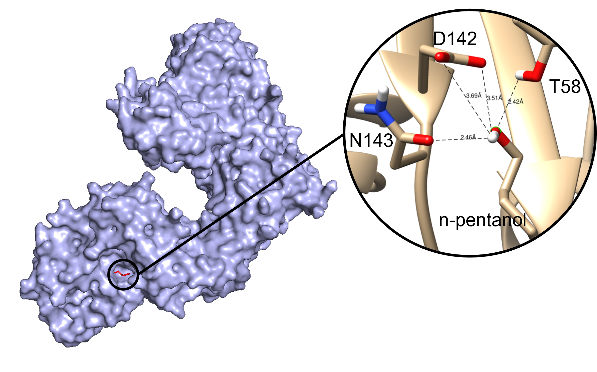** |
| --- |
| **B**  **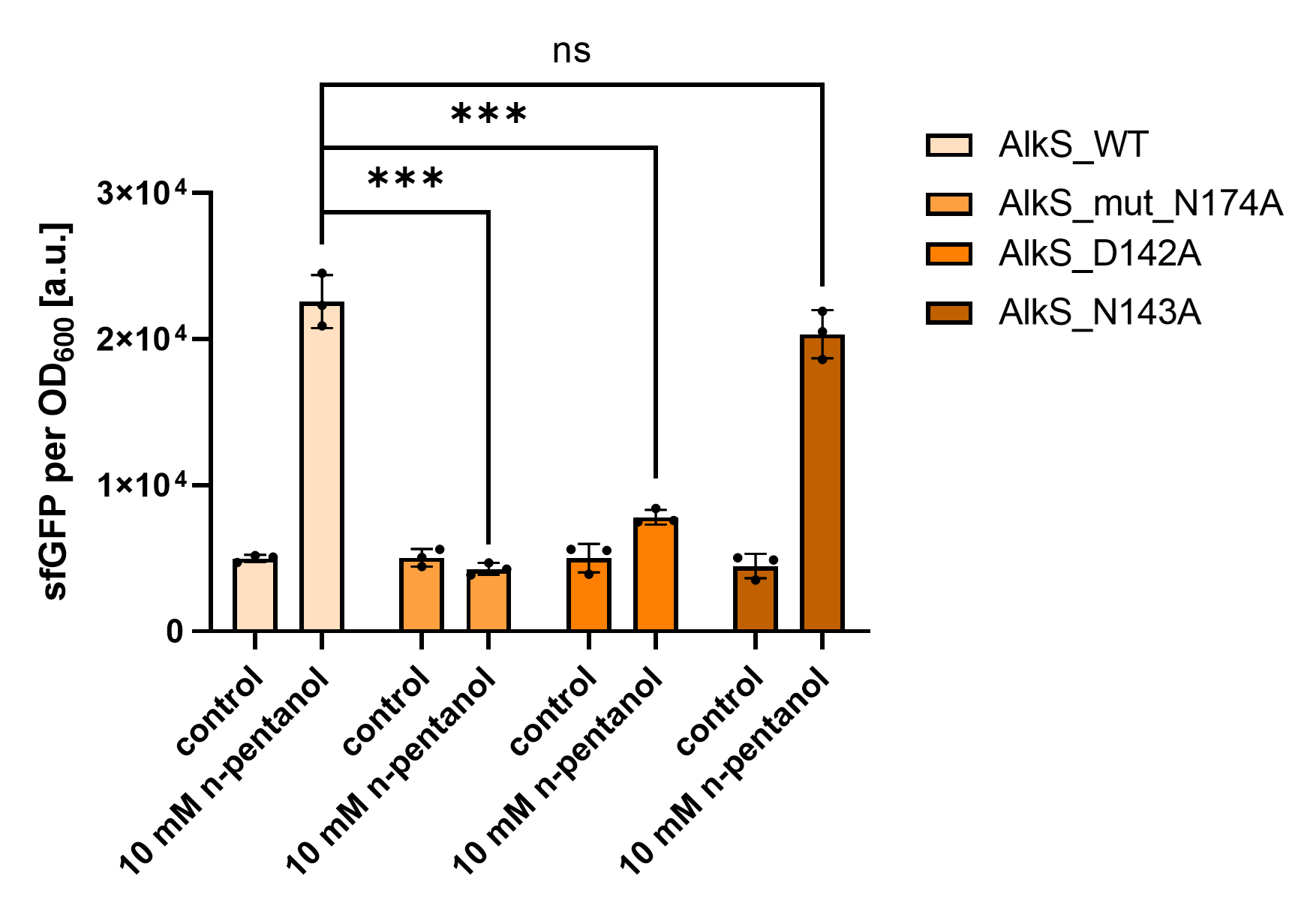** |
| **C**  **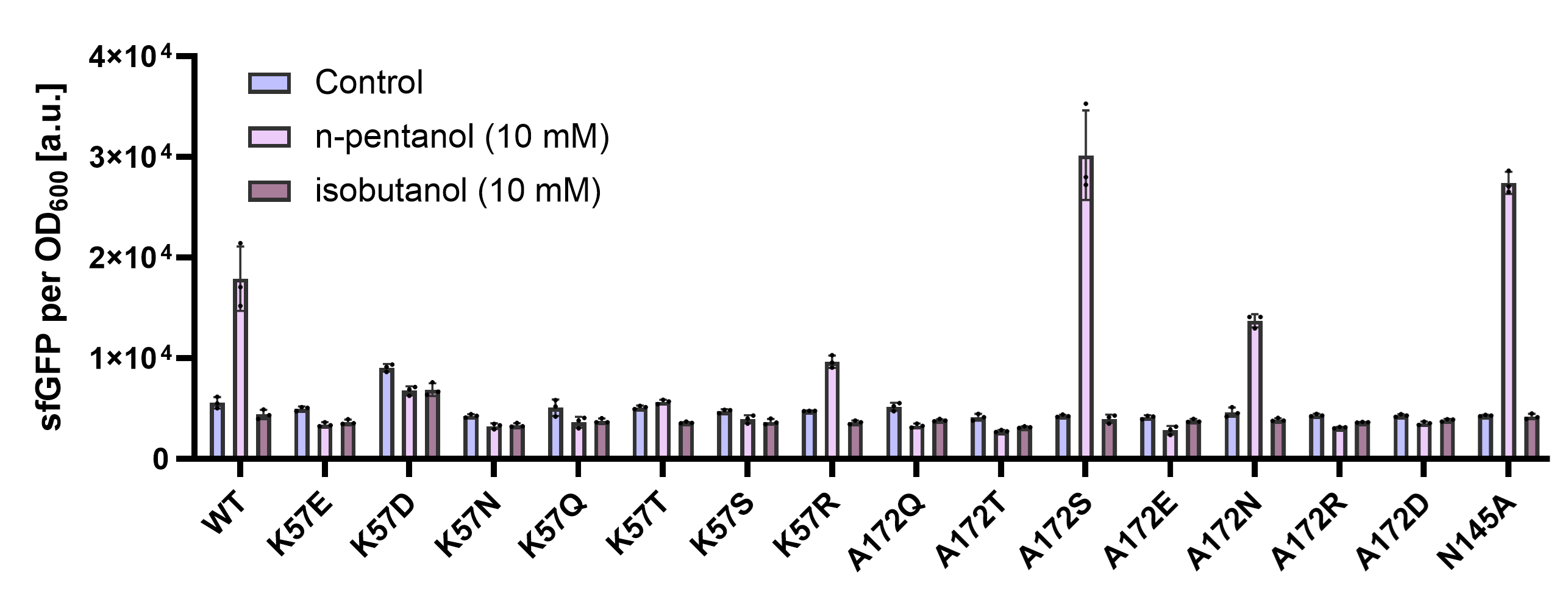** |
| Figure S2. A. Schematic diagram of docking simulations between AlkS and n-pentanol. AlkS is shown in purple, n-pentanol is highlighted in red. Predicted binding pocket and potential key residues (T58, D142 and N143) that could form hydrogen bonds with n-pentanol are shown in black circle. B. Alanine-mutation results for speculated key residues (T58, D142 and N143) during the binding between AlkS and n-pentanol. C. Response of single-point AlkS mutants to isobutanol and n-pentanol. The experiments were carried out in triplicate with error bars representing the standard deviation from the mean. |

| S3. Fluorescence-activated Cell Sorting Results  **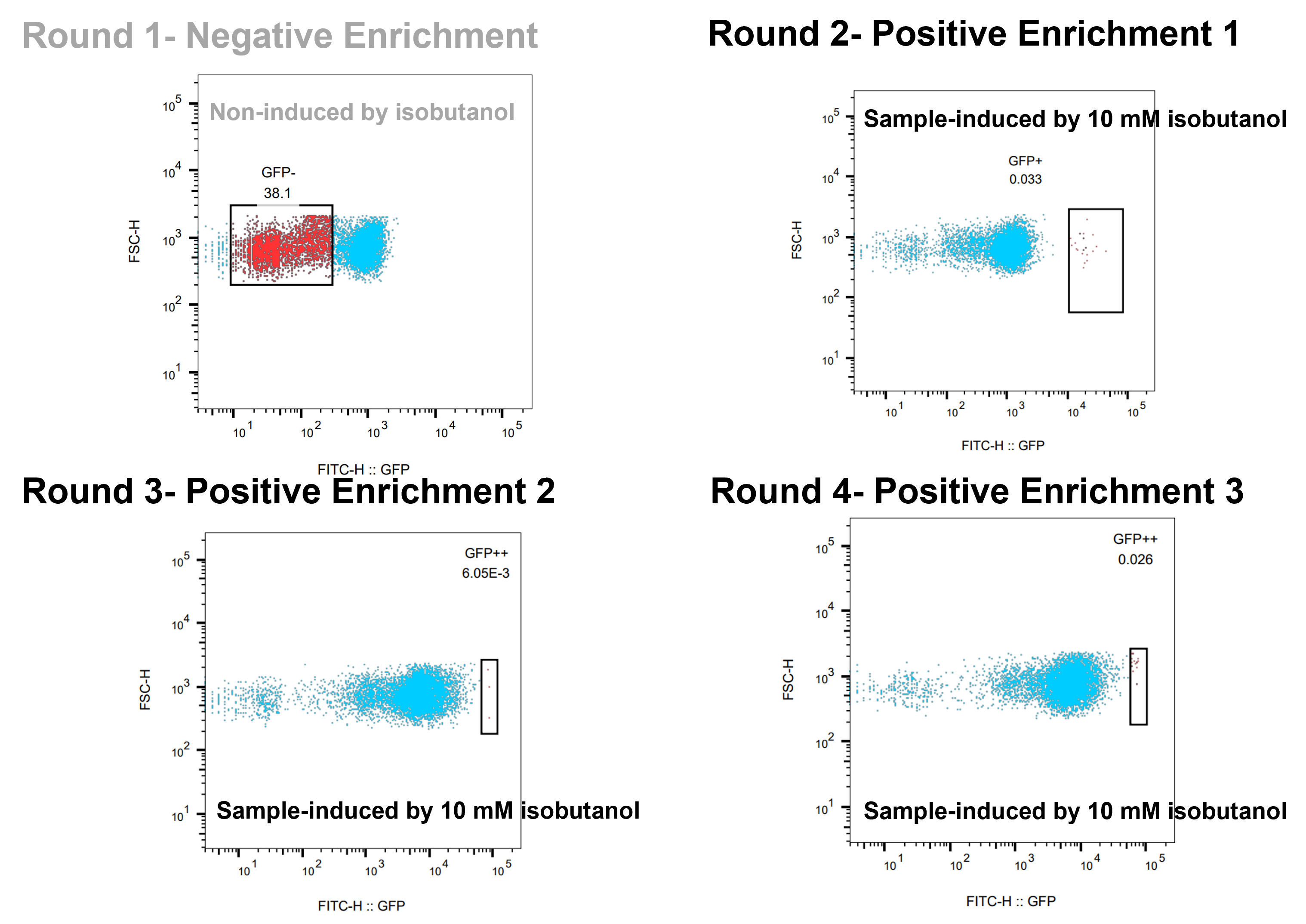** |
| --- |
| Figure S3. Fluorescence-activated Cell Sorting Results (4 rounds with the first negative control). |

S4. Strains, plasmids and primers

## Table S1. Strains applied in this study.

| **Strains** | **Description** | **Source** |
| --- | --- | --- |
| BW25113 | K 12 derivatives. F-, *Δ(araD-araB)567*, *ΔlacZ4787(::rrnB-3),* λ-, rph-1, *Δ(rhaD-rhaB)568*, *hsdR514* | (Datsenko & Wanner, 2000) |
| BW25113-*P_alkS_-alkS-P_alkB_-sfgfp* | Carries *pSB50C7* | This study |
| BW25113-*P_alkS_-alkS (D_e-p_)-P_alkB_-sfgfp*-1p | Carries *pSB50C7 (D_e-p_)*. The sensing module and reporter module are on the same plasmid. | This study |
| BW25113-*P_alkS_-alkS (D_e-p_)*/*P_alkB_-sfgfp* | Carries *pACYC184-P_alkS_-alkS* *(D_e-p_)* and *pUC19-P_alkB_-sfgfp* | This study |
| BW25113- *P_alkS_-alkS-P_alkB_-sfgfp* | Carries *pACYC184-P_alkS_-alkS* and *pUC19-P_alkB_-sfgfp* | This study |
| BW25113-*P_alkS_-alkS_mut-P_alkB_-sfgfp* | Carries *pACYC184-P_alkS_-alkS (mut)* and *pUC19-P_alkB_-sfgfp* | This study |
| BW25113-*P_alkS_***-***alkS_mut_A172S-P_alkB_-sfgfp* | Carries *pACYC184-P_alkS_-alkS (mut)-A172S* and *pUC19-P_alkB_-sfgfp* | This study |
| BW25113-*PalkS-alkS_mut-PalkB_mut-sfgfp* | Carries *pACYC184-P_alkS_-alkS (mut)* and *P_alkB_ (mut)-sfgfp* | This study |
| BW25113- *PalkS-alkS_mut_A172S-PalkB_mut-sfgfp* | Carries *pACYC184-P_alkS_-alkS (mut)-A172S* and *P_alkB_ (mut)-sfgfp* | This study |

Table S2. Plasmids applied in this study.

| **Plasmid** | **Characteristics** | **Source** |
| --- | --- | --- |
| pSB50C7 | *P_alkS_-alkS-P_alkB_-sfgfp* biosensor plasmid. p15A replication origin, medium copy number, Cm^R^ | Lab stock |
| pACYC184-PalkS-alkS | *P_alkS_-alkS* biosensor plasmid. p15A replication origin, low copy number, Cm^R^ | This study |
| pACYC184-PalkS-alkS (De-p) | *P_alkS_-alkS (D_e-p_)* biosensor plasmid. p15A replication origin, low copy number, Cm^R^ | This study |
| pACYC184-PalkS-alkS (mut) | *P_alkS_-alkS (mut)* biosensor plasmid. p15A replication origin, low copy number, Cm^R^ | This study |
| pACYC184-PalkS-alkS-T58A, D142A, or N143A | *P_alkS_-alkS (alanine scanning)* biosensor plasmid, including T58A, D142A or N143A mutation in *alkS*. p15A replication origin, low copy number, Cm^R^ | This study |
| pACYC184-PalkS-alkS-N145A, K57R, K57S, K57T, K57N, K57Q, K57D, K57E, A172R, A172S, A172N, A172Q, A172D, A172E, A172T, or N174A | *P_alkS_-alkS (mut_site-directed mutagenesis)* biosensor plasmid, including N145A, K57R, K57S, K57T, K57N, K57Q, K57D, K57E, A172R, A172S, A172N, A172Q, A172D, A172E, A172T, N174A mutation in *alkS*. p15A replication origin, low copy number, Cm^R^ | This study |
| pUC19-PalkB (mut)-sfgfp | *P_alkB_ (mut)-sfgfp* biosensor plasmid, with sequence change in the core promoter region. colE1 replication origin, low copy number, Amp^R^ | This study |

Table S3. DNA primers used in this study.

| **Primer** | **Nucleotide sequences (5’ to 3’)** |
| --- | --- |
| **Alanine Scanning** | |
| T58A-fw | AGCAACGGcTTTCCCATATCCAGGTGG |
| T58A-rv | GGAAAgCCGTTGCTCTTGCGTTCG |
| D142A-fw | TAATATTAgCCAAACAAACTAGCGTTTCTATCTCGTTATTCG |
| D142A-rv | GCTAGTTTGTTTGGcTAATATTAATCATGACTTAGACTTGCCG |
| N143A-fw | GATTAATAgcATCCAAACAAACTAGCGTTTCTATCTCGTTATTCG |
| N143A-rv | GTTTGTTTGGATgcTATTAATCATGACTTAGACTTGCCGTTGTTG |
| **Site-directed Mutagenesis** | |
| A172D-fw | GTATTGCCatcAACTGCAAACCTGATATTTTTTGGTG |
| A172D-rv | GCAGTTgatGGCAATACAATAAAAGGGTTCTCGC |
| A172E-fw | GTATTGCCttcAACTGCAAACCTGATATTTTTTGGTG |
| A172E-rv | GCAGTTgaaGGCAATACAATAAAAGGGTTCTCG |
| A172N-fw | GTATTGCCgttAACTGCAAACCTGATATTTTTTGGTG |
| A172N-rv | GCAGTTaacGGCAATACAATAAAAGGGTTCTCGCAG |
| A172Q-fw | GTATTGCCttgAACTGCAAACCTGATATTTTTTGGTG |
| A172Q-rv | GCAGTTcaaGGCAATACAATAAAAGGGTTCTCGC |
| A172R-fw | GTATTGCCtcgAACTGCAAACCTGATATTTTTTGGTG |
| A172R-rv | GCAGTTcgaGGCAATACAATAAAAGGGTTCTCGC |
| A172S-fw | GTATTGCCtgaAACTGCAAACCTGATATTTTTTGGTG |
| A172S-rv | GCAGTTtcaGGCAATACAATAAAAGGGTTCTCGC |
| A172T-fw | GTATTGCCtgtAACTGCAAACCTGATATTTTTTGGTG |
| A172T-rv | GCAGTTacaGGCAATACAATAAAAGGGTTCTCGC |
| K57D-fw | CAACGGTatcCCCATATCCAGGTGGGGCTC |
| K57D-rv | ATATGGGgatACCGTTGCTCTTGCGTTCGAGT |
| K57E-fw | CAACGGTttcCCCATATCCAGGTGGGGCTC |
| K57E-rv | ATATGGGgaaACCGTTGCTCTTGCGTTCGAGT |
| K57N-fw | CAACGGTgttCCCATATCCAGGTGGGGCTC |
| K57N-rv | ATATGGGaacACCGTTGCTCTTGCGTTCGAGT |
| K57Q-fw | CAACGGTttgCCCATATCCAGGTGGGGCTC |
| K57Q-rv | ATATGGGcaaACCGTTGCTCTTGCGTTCGAGT |
| K57R-fw | CAACGGTtcgCCCATATCCAGGTGGGGCTC |
| K57R-rv | ATATGGGcgaACCGTTGCTCTTGCGTTCGAGT |
| K57S-fw | CAACGGTtgaCCCATATCCAGGTGGGGCTC |
| K57S-rv | ATATGGGtcaACCGTTGCTCTTGCGTTCGAGT |
| K57T-fw | CAACGGTtgtCCCATATCCAGGTGGGGCTC |
| K57T-rv | ATATGGGacaACCGTTGCTCTTGCGTTCGAGT |
| N145A-fw | CAAGTCTAAGTCATGagcAATATTATCCAAACAAACTAGCG |
| N145A-rv | GATAATATTgctCATGACTTAGACTTGCCGTTGTTGCAC |
| N174A-fw | CAGGCgctACAATAAAAGGGTTCTCGCAGC |
| N174A-rv | TTATTGTagcGCCTGCAACTGCAAACCTGATA |
| **Error-prone PCR** | |
| alkS-DE-fw | GTGACATGCATATTTGTTGCTATTTGTTTGTT |
| alkS-DE-rv | GCTGGCGCGAGAATAGCATAATG |
| alkS-DE-vec-fw | CATTATGCTATTCTCGCGCCAGC |
| alkS-DE-vec-rv | AACAAACAAATAGCAACAAATATGCATGTCAC |
| **Promoter Engineering** | |
| palkB-35-fw | GTAGTTGacaCAAGCGTCCGATTAGCTCAGG |
| palkB-35-rv | GCTTGtgtCAACTACACCTACGGGTAGTTTAAG |
| palkB-10-fw | AAGCGTCCGATTAGCTataaTTTAAGATGTCGAGAGTGAGAGTG |
| palkB-10-rv | CACTCTCGACATCTTAAAttatAGCTAATCGGACGCTT |
